# Editorial: Role of Stem Cells in Skeletal Muscle Development, Regeneration, Repair, Aging, and Disease

**DOI:** 10.3389/fnagi.2016.00095

**Published:** 2016-04-28

**Authors:** Pura Muñoz-Cánoves, Jaime J. Carvajal, Adolfo Lopez de Munain, Ander Izeta

**Affiliations:** ^1^Cell Biology Group, Department of Experimental and Health Sciences, Pompeu Fabra UniversityBarcelona, Spain; ^2^CIBERNED, Instituto de Salud Carlos IIIMadrid, Spain; ^3^ICREABarcelona, Spain; ^4^Centro Nacional de Investigaciones Cardiovasculares (CNIC)Madrid, Spain; ^5^Molecular Embryology Team, Centro Andaluz de Biología del DesarrolloSeville, Spain; ^6^Neuroscience Area, Instituto Biodonostia, Hospital Universitario DonostiaSan Sebastian, Spain; ^7^Department of Neurosciences, Faculty of Medicine and Dentistry, University of the Basque CountrySan Sebastian, Spain; ^8^Department of Neurology, Hospital Universitario DonostiaSan Sebastian, Spain; ^9^Tissue Engineering Laboratory, Bioengineering Area, Instituto Biodonostia, Hospital Universitario DonostiaSan Sebastian, Spain

**Keywords:** satellite cells, pericytes, myogenesis, centenarians, rejuvenation, signaling pathways, muscle atrophy, muscle dystrophies

Skeletal muscle is a highly dynamic and plastic tissue, able to modify its intrinsic size or strength following electric impulse, mechanical loading, or diet. Several muscular dystrophic disorders have been characterized but the development of therapies, although promising, is still at an early phase. Muscle dysfunction is not restricted to dystrophic patients; during aging, there is a gradual loss of muscle function that results in a significant negative impact on the individual's health, increasing fall and lesion risks, loss of mobility and independence, and associated elevation of morbidity and mortality. This loss of muscle has an estimated prevalence between 5 and 13% among 60–70 year old individuals, increasing to 11–50% in those over the age of 80 (Morley, 2008). According to the WHO, the expected number of individuals over 65 years old by the year 2050 will be around 1.5 billion (WHO, 2015); by extrapolation, this suggests that over 150 million patients will suffer from muscle wasting disorders associated with aging.

The vertiginous development of the stem cell therapy and cellular reprogramming fields will eventually result in the emergence of cell replacement therapies to treat a wide range of pathologies. In skeletal muscle, the satellite cell—long regarded as a heterogeneous cell population—is intimately linked to muscle physiology and regeneration processes (Chang and Rudnicki, 2014; Dayanidhi and Lieber, 2014; Snijders et al.). The activation of these muscle-specific stem cells is essential for injury healing, maintenance of muscle strength and tone, and delayed onset of age-related sarcopenia.

In this issue, eight review and eight original research articles on this Research Topic summarize recent progress and current challenges of the muscle biology field in aging and disease.

## Role of stem cells in skeletal muscle development

Stem cells have a key role during the sequential stages of embryonic muscle development, as demonstrated through many decades of work using a multitude of animal models (Brand Saberi, 2015). In this issue, Knight and colleagues develop a novel *pax7a:eGFP* transgenic zebrafish injury-model in which they demonstrate quantitatively that migration of GFP+ cells upon injury depends on the specific characteristics of the induced damage and on the embryonic developmental stage (Knappe et al.). This suggests that muscle-resident *pax7a*-expressing cells may function differently depending on experimental conditions; unraveling these diverse functions represents a challenge and has important implications for muscle repair and regeneration studies.

Muscular dystrophies target head and trunk muscles differently, indicating that satellite cells, which drive muscle regeneration, may have molecular functional differences in each muscle tissue. Dietrich and colleagues report on the regulation of Pax7-expressing head muscle stem cells by comparing chicken, mouse, frog and zebrafish models (Nogueira et al.). They postulate a somewhat counterintuitive mechanism for the emergence of craniofacial muscle stem cells, whereby *de novo* expression of Pax7 is acquired later than the cellular commitment to the myogenic fate, which is established by expression of the myogenic regulatory factors (MRFs). Guardiola and colleagues generate a novel mouse model to better understand the importance of Cripto—a known regulator of vertebrate embryogenesis—during postnatal skeletal muscle regeneration (Prezioso et al.). When overexpressed in adult satellite stem cells, Cripto promotes myogenic commitment and fusion/differentiation in a time-dependent manner, improving muscle regeneration upon acute injury. Availability of the *Tg:Cripto* model will be instrumental for the future development of pharmacological approaches that target this important pathway.

## Role of stem cells in skeletal muscle regeneration and repair

A number of epigenetic modifications are known to be key in the regulation of satellite cell function (Giordani and Puri, 2013; Dumont et al., 2015; Parker; Segales et al., 2015; Sousa-Victor et al., 2015). Epigenetic marks on chromatin can be modified, modulated, written and/or erased by the action of growth factors, inflammatory signals, cellular redox status, developmental pathway switches, and/or mechanical stimuli. The potential reversibility of most of these epigenetic modifications provides an attractive targeting approach for pharmacological manipulation. Palacios and colleagues exhaustively review what is known about the epigenetic impact of the signaling microenvironment upon satellite cell chromatin during adult regenerative myogenesis (Brancaccio and Palacios). Complementarily, Suelves and colleagues review DNA methylome (DNA methylation signature) dynamic changes during the processes of commitment and differentiation of skeletal muscle, driven by the activation of the MRFs, and address what is known about the methylome status in pathological and aging processes (Carrio and Suelves).

Satellite cells are not the only stem cells residing in skeletal muscle, and other progenitors and immune cell infiltrates can influence this tissue's regenerative potential (Burzyn et al., 2013; Mozzetta et al., 2013; Pannerec et al., 2013; Farup et al., 2015; Kostallari et al., 2015). Two articles in this issue summarize the role of non-satellite stem cells in muscle biology as well as their potential use in cell-based therapies. Delbono and colleagues review the different roles of muscle-resident perivascular cells in skeletal muscle physiology, defining two distinctive types of pericytes. Their effects are linked—but not limited—to positive outcomes, such as support for muscle regeneration, reinnervation, and *de-novo* vessel formation and negative outcomes, including unwanted differentiation, fibrosis, fat accumulation, and heterotopic ossification (Birbrair et al.). The article serves as a powerful warning for those eager to push the early implementation of stem cell therapies, and highlights the need to deepen our knowledge on the activation and integration of transplanted stem cells, as these will not contribute to the regeneration processes of the targeted organ in all therapeutic approaches and could indeed be detrimental for the patient.

In a distinct contribution, Forcales puts forward the case for the closely related adipose-derived mesenchymal stem cell (ASC) to be used for muscle regenerative therapies. The article presents a collective view of ASC's myogenic differentiation, engraftment and functional assessment protocols as reported by different research groups. Since these cells have shown good safety records but poor efficacy when transplanted, the author advocates for enhanced enrichment, expansion and manipulation of ASC-derived myogenic progenitors to improve clinical outcomes.

## Role of stem cells in skeletal muscle aging

Centenarians are the paradigm of healthy aging, and their exceptional longevity and physical fitness must somehow be related to genetic make-up. The identification of genetic polymorphisms linked to muscle mass and function maintenance in the old age should be advantageous for the prevention of age-related sarcopenia. In this long road, discarding many potential candidates is also important. Lucia and colleagues study the functional significance (i.e., whether they affect the muscle transcriptional levels of the gene) of three candidate SNPs in independent human cohorts. Although, they detect correlation with transcriptional levels of the genes in close proximity to the SNPs, they do not find association with exceptional longevity (Fuku et al.). These findings are relevant and intriguing because these SNPs, which are associated with lean body mass, cardiorespiratory fitness and better physical performance, nevertheless do not seem to be related necessarily to an increase in longevity.

Another theoretical therapeutic approach gaining momentum is directed toward the rejuvenation of the stem cell niche through its exposure to a “younger environment.” The identity of the particular systemic factors mediating this promising therapeutic possibility is under debate (Li and Izpisua Belmonte, 2014; Sinha et al., 2014; Sousa-Victor et al., 2014, 2015; Brun and Rudnicki, 2015; Egerman et al., 2015; Rodgers and Eldridge, 2015). Latella and colleagues provide an interesting review of some recent breakthrough studies on cell-intrinsic vs. cell-extrinsic mechanisms underlying satellite cell regenerative decline with aging (Madaro and Latella). They advocate for new studies to further explore the relationship between DNA damage, p38 and p16^INK4a^ signaling pathways on this age-associated decline.

The control of fibroblast growth factor (FGF) signaling by Spry1 is altered with aging and affects satellite cell quiescence maintenance (Chakkalakal et al., 2012; Tajbakhsh, 2013). Yablonka-Reuveni et al. show that only Fgfr1 and Fgfr4 receptors are expressed in satellite cells; to better understand their role in adult muscle regeneration upon injury, the authors analyse the phenotype after Fgfr1 ablation. They show that although FGF2-driven mitogenic response in satellite cells is virtually absent, this does not affect muscle regeneration, possibly due to the existence of compensatory mechanisms.

## Role of stem cells in diseases of the skeletal muscle

Muscular dystrophies are among the most common and severe diseases affecting skeletal muscle homeostasis and function. While dystrophies share many common characteristics—most being linked to the dystrophin-associated protein complex—a distinct feature, used in diagnosis, is the differential effect of the particular mutation on the various skeletal muscle groups. Satellite stem cells are considered a notoriously heterogeneous cell population (Motohashi and Asakura; Dumont et al., 2015). Pavlath and colleagues review what is known on skeletal muscle stem cell biology and compare satellite cells from eight different muscle groups and the distinct extent of affection of the same muscles in muscular dystrophies (Randolph and Pavlath). The authors highlight the importance of further advancing our knowledge on the biology of muscle derived satellite cells from non-limb muscles, which have somehow been neglected until now. Following this theme, Marazzi and colleagues analyze variations on the support role provided by interstitial progenitor cells (“PICs”) to muscle regeneration in an effort to further characterize the satellite stem cell niche in extraocular muscles (Formicola et al.). Interestingly, they observe an increase in the number of PICs in both aged and dystrophic animals. Because the maintenance of a high number of PICs positively supports myogenesis, they conclude that that the extraocular-specific increase of PIC numbers might underlie the resistance to dystrophy by this muscle subset.

Biressi and Gopinath challenge the current thinking on the connections between satellite stem cells and loss of muscle mass, otherwise known as muscle wasting or atrophy. They review the signaling pathways implicated and conclude that multiple cause-related atrophies coexist within a single clinical entity and that this may hinder the precise targeting required for the development of novel therapeutic approaches.

Fittingly with the proposed topic, Lopez de Munain and colleagues review the available evidence to classify Myotonic dystrophy type 1 as a progeroid syndrome (Mateos-Aierdi et al.). A number of the so-called “hallmarks of aging” (Lopez-Otin et al., 2013) are detected in these patients whose disease is possibly associated with satellite cell dysfunction. The article proposes some of the research avenues that might be worth exploring in the future.

Several microRNAs (miRs) have been implicated in muscle stem cell function and homeostasis; their role on muscle dysfunction and their associated disease has also been extensively investigated (Rosales et al., 2013; Dey et al., 2014; Hindi and Kumar, 2016). Musaró and colleagues locally overexpressed the anabolic growth factor insulin-like growth factor 1 (IGF-1) in Duchenne muscular dystrophy (*mdx*) muscle and studied modulation of the miR signature, finding major changes in miR-206 and miR-24 levels, that contributed to muscle differentiation (Pelosi et al.). This research links the activation of anabolism by the IGF-1 pathway in skeletal muscle with changes in essential miRNA molecules never implicated in this process before.

Finally, in McArdle disease, a genetic disorder of skeletal muscle metabolism, patients struggle to perform resistance exercise due to increased risk of severe rhabdomyolysis, which may develop into acute tubular necrosis, renal failure, and severe low blood pressure. In a clinical study, Lucia and colleagues propose a novel resistance-training program in adult McArdle patients that was well-tolerated according to clinical assessments. The preliminary data support the possibility of a therapeutic intervention involving regular participation in strength training programs, with the potential to improve muscle mass, strength and clinical outcome of McArdle patients (Santalla et al.).

## Future prospects

The field of skeletal muscle stem cell biology has exploded in the past decade, although the advance experienced in our knowledge of the function, origin, renewal, gene regulation, epigenetics, aging, and senescence of these remarkable cells, only represents a small step toward our full understanding of their abilities and therapeutic potential. Many aspects of these cells are still puzzling and would require a combined effort to unravel their many secrets. We still lack comparative metabolomic and transcriptomic analysis between cells of various origins and muscle groups that may provide a more focused view on the inherent properties of different muscles and their resistance to disease; the explosion of single-cell “omic” approaches will provide an unexpected wealth of new data and insight into the biology of these cells. Another key area of present and future development is to develop pharmacological or cell-based therapeutic approaches to transcriptionally regulate satellite cells so that they become amenable for clinical manipulation and therapeutic deployment. In this quick journey from development to adulthood, from regeneration to disease, we hope the reader finds some enlightenment on the multiple facets of satellite cell biology, which is not free of discrepancies. In all, an increased understanding of muscle biology makes us even more conscious of the limits of our knowledge. Or, as Walt Whitman wrote “*Do I contradict myself? Very well, then, I contradict myself; (I am large-I contain multitudes.).”*

## Author contributions

AI drafted the manuscript that was amended and approved by all authors, acting as co-Editors of the Research Topic.

## Funding

Work in our labs was supported by grants from Instituto de Salud Carlos III (ISCIII; PI13/02172 to AI and PI14/7436, CIBERNED and Fundación Isabel Gemio to ALM); from SAF2012-38547, SAF2015-67369-R, AFM, Marató-TV3, E-Rare/Eranet, MDA to PM; JC received support from the Spanish Ministry of Science (BFU2014-54194-P), the AFM and the European Commission (CIG-303904). AI was supported by the Programa I3SNS (CES09/015) from ISCIII and by Osakidetza-Servicio Vasco de Salud (Spain).

### Conflict of interest statement

The authors declare that the research was conducted in the absence of any commercial or financial relationships that could be construed as a potential conflict of interest.
